# A Historical and Theoretical Review of Cognitive Behavioral Therapies: From Structural Self-Knowledge to Functional Processes

**DOI:** 10.1007/s10942-018-0292-8

**Published:** 2018-04-13

**Authors:** Giovanni M. Ruggiero, Marcantonio M. Spada, Gabriele Caselli, Sandra Sassaroli

**Affiliations:** 1 “Psicoterapia Cognitiva e Ricerca” Cognitive Psychotherapy School and Research Center, Milano, Foro Buonaparte 57, 20121 Milan, Italy; 2 “Studi Cognitivi” Cognitive Psychotherapy School and Research Center, Foro Buonaparte 57, 20121 Milan, Italy; 3 Sigmund Freud University, Ripa di Porta Ticinese 77, 20143 Milan, Italy; 4 Sigmund Freud University, Freudplatz 1, Messestraße 1, 1020 Vienna, Austria; 5 Division of Psychology, School of Applied Sciences, London South Bank University, 103 Borough Road, London, SE1 0AA UK

**Keywords:** Cognitive therapies, Constructivism, Functionalism, Processes, Self-belief, Structuralism

## Abstract

This paper critically examines the historical conceptualization of cognitive behavioral psychotherapy approaches (CBT) as a direct clinical counterpart of the cognitive revolution. The main “second wave” cognitive psychotherapies, either standard cognitive therapy (CT) or constructivist, in spite of their differences, share a common conceptualization of psychopathological factors as superordinate structural cognitive content belonging to the self: self-beliefs, self-schemata, personality organizations and so on. On the other hand, rational emotive behavior therapy (REBT) is an exception given that in REBT self-knowledge is not the core psychopathological tenet, being rather a derivate mechanism. Moreover, in non clinical cognitive science cognition is conceived as a regulatory function that operates retroactively and not in a hierarchically super- ordered fashion centered on the self. A historical review suggests that in both CT and constructivist model the structuralistic model of self-centered cognition may have emerged for both cultural and scientific reasons: self-centered cognitive models may be more readily understandable to clinicians as they allow for a straightforward identification of operationalizable self-beliefs. The emergence of new “third wave” process-centered CBT approaches may represent a comeback to functionalism, where cognition is considered again a regulatory function and not a structure. In addition, REBT’s interest in dysfunctional evaluations not focused on the self presaged this clinical and scientific turning point toward functionalism.

## The Role of Self-Knowledge in the Clinical Theory of Cognitive Therapies

The birth of cognitive behavioral psychotherapy (CBT) approaches has often been described as the clinical equivalent of the cognitive revolution which took place in the field of scientific psychology thanks to Chomsky (1959), Miller et al. (1960), Newell et al. (1958) and many others. The revolution consisted of the addition of a cognitive mediator interposed between environmental triggers and behavioral responses. This cognitive mediator would be organized in terms of self-schemata which play a structural role: self-schemata would provide guide, consistency, coordination, and integration to mental states (Neisser 1967; Markus 1977).

Sometimes this revolution that led to CBT approaches is also called “second wave” since the cognitive mediator would have been absent in the “first wave” behavioral model (Hayes 2004). Ellis’ rational emotive behavior therapy (REBT; Ellis 1955, 1962; Ellis and Grieger 1986) and Beck’s cognitive therapy (CT; Beck 1963, 1964, 1976; Beck et al. 1979) were the champions of the “second wave” being the first to propose clinical counterparts to the cognitive revolution. They were followed by other, less famous theorists, including Lazarus (1976), Mahoney (1974, 1991, 1995a, b, 2003), Meichenbaum (1977), Goldfried and Davison (1976) and many others. At a later time, “second wave” bifurcated into a “rationalist” approach which included both Beck’s CT and Ellis’ REBT and conceived cognition as a conscious computational knowledge and a “constructivist” approach which viewed cognition as a hermeneutical, emotionally laden, and “tacit” knowledge stemming from human relationships (Mahoney 1995b; Guidano and Liotti 1983). Finally, the so-called “third wave” would have happily concluded the story by integrating functional processes in the overriding model of CBT approaches (Hayes and Hofman 2018).

**Fig. 1:**
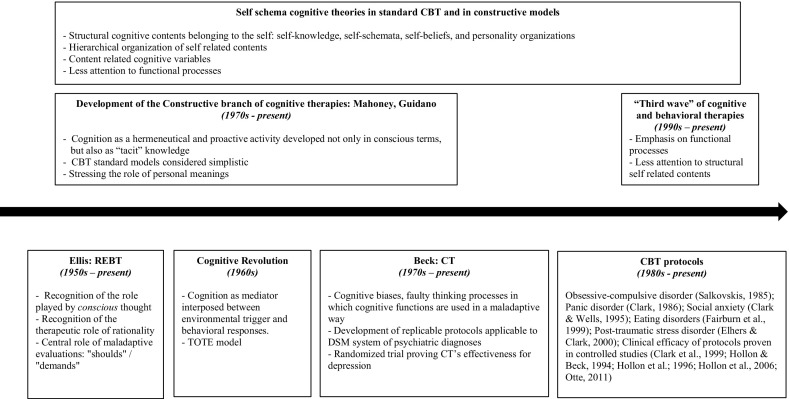
The development of cognitive therapies

However, we argue for a different narrative of the development of CBT in which (1) at the beginning, cognitive science viewed cognition not as a structural mediator organized in terms of self-centered contents of knowledge but as functionalist retroactive processes which were already partially present in the behavioral “first wave” and foreran the “third wave” process centered models; (2) despite the supposed divergences between “rationalist” and “constructivist” approaches of the “second wave” both converged toward a structuralist conception of self-knowledge that had roots not only in cognitive science but also in some psychodynamic models; (3) within the “second wave” REBT is an exception given that it does not consider self-knowledge a structuralist psychopathological tenet; (4) “third wave” process centered models retrieve the functionalist models of cognitive science and reject the structuralist self-psychology of the “second wave” and may be classified in top–down and bottom–up models, in relation to which functions they prefer to confer the role of strategic bottleneck to be targeted in therapy. Figure 1 represents this differing narration. This paper aims to illustrate this view in detail.

The theoretical models of the cognitive revolution allowed for the abandonment the stimulus–response behavioral model and replaced it by using the so-called Test-Operate-Test-Exit or TOTE model described by Miller et al. (1960), which is the basic unit of cognitive functioning. The TOTE model proposes that in a cognitive behavioral sequence individuals plan a goal and perform a test (T) in order to determine whether the goal has been accomplished. When individuals do not accomplish the goal, they cognitively conceive and behaviorally perform operations (O) in order to achieve the goal. After that, individuals perform the test (T) again, and exit (E) occurs if the goal is achieved. Otherwise, the process repeats. For example, a perception of threat and fear (T) generates an escape reaction (O) that continues to be used (T) until the disappearance of the threat.

When re-examing in depth the TOTE model, it is clear that cognitive function is not conceived as nowadays as a structural mediator schema interposed between trigger and response but more aptly as a feedback which controls and acts retroactively performing operations (O) not on inert environmental triggers but on mental evaluative states not coincidentally called “test” (T), correcting and regulating them until the mind would accomplish the desired outcome called “exit” (E). Therefore, the O function does not work as a mediational cognitive operator but rather as a retroactive executive agent providing control feedbacks on mental states, a function which is more metacognitive than properly cognitive, being a second-order regulation -within the mind itself- of mental states by mental processes and not a first-order cognitive evaluation of an object to know (Wells and Mathews 2015, p. 31; Williams et al. 1988). In addition, in these early models, there was no mention of cognitive content focused on the typical self-belief and self-schemata of later models (for example as heralded in the “schema theory” postulated in mature “standard” cognitive psychotherapy).

However, it is apparent that over time in cognitive science emerged a “schema theory” focused on self-centered mental variables which play a mediating role. This phenomenon dates back to the publication of models by Neisser (1967) and especially by Markus (1977). Additionally, we cannot forget to quote the influence of Bandura’s model of self-efficacy (Bandura 1977, 1988). These models privileged the role of structural cognitive mediators organized in the form of knowledge of the self (*self*-*knowledge*), patterns of the self (*self*-*schemata*) and beliefs about the self (*self*-*belief*).

Cognitive self schemata would be stable and integrated organizations of knowledge which would summarize an array of information and experience in friendly manageable packages (Markus and Sentis 1982). Among schemata, self-schemata would be hierarchically superordinate and unique in that they integrate and summarize a person’s thoughts, feelings, and experiences. This means that any attempt to organize one’s own behavior in a particular domain would always result in the formation of cognitive structures about the self. Self-schemata are established in domains that the person values, including physical characteristics, social roles, personality traits, and areas of particular interest and skill (Markus and Nurius 1986).

However, in the clinical field of CBT, the preference for self-schemata and self-knowledge was not a later development but prevailed from the beginning. This different evolution highlights a real difference in historical development between cognitive science and CBT approaches. Hollon and DiGiuseppe (2010), in their exhaustive historical account, have confirmed that the cognitive revolution in cognitive psychology and CBT did not appear to effect each other.

Beck’s CT model explained emotional disorders using cognitive biases, faulty thinking processes in which cognitive functions are used in a maladaptive way. In turn, cognitive biases were packed into conscious negative schemata focused on the self, the world/environment and the future (CT’s cognitive triad). Over time, self-schemata won a prevailing role within the cognitive triad of CT (Wells and Mathews 1994, p. 2). As mentioned earlier, this final prevalence of self-schemata theory is also attributable to the influence of the clinical applications of Bandura’s seminal work on self-efficacy (Maddux and Kleiman 2012). Summing up, mature cognitive models elevate self-judgment to foundational levels in explaining emotional stability. Positive self-judgments about being able to manage and control events and their own emotional reactions are seen as largely responsible for their emotional well-being and daily life efficacy. Negative self-judgments are what make them depressed or anxious (Williams 1996).

## Psychodynamic Influence on Self-Knowledge

However, it is possible that Beck’s CT switch from cognitive biases to negative self-schemata depended not only on the parallel switch operated by Bandura, Neisser, and Markus in cognitive science, but also by Beck’s psychotherapeutic background. Initially, Beck had psychodynamic training in the field of American *ego*-*psychology* developed by Anna Freud (1936/1966) and Hartmann and Loewenstein (Hartmann 1964; Hartmann and Loewenstein 1964). In this psychodynamic paradigm, it was supposed that the human mind owns ego functions can be either the result of adaptive and normal development of mental capacities or may be influenced by conflicted aggressive and libidinal impulses (Rosner 2014a, b). In short, the ego plays a key organizational role in mental activity in *ego*-*psychology*, both in normality and in psychopathological states which seems similar to the structural role played by the self in the cognitive clinical psychology inspired by Beck.

Beck was trained at the Philadelphia Institute of the American Psychoanalitic Association. Rosner (2014a, b) recounts that he abruptly switched to the cognitive paradigm at some point in the 1960s during his research activity, after he failed to confirm the psychodynamic hypothesis of depression as an outcome of anger and aggression drives. Beck’s clinical development, however, appears more uneven. It is true that after his first doubts about the psychoanalytic model, Beck began studying the clinical and theoretical works of Ellis, Lazarus, Mahoney, and Davidson, pioneers of the cognitive clinical switch. Yet at the same time Beck continued to partially consider himself a follower of ego-psychology, the neo-Freudian psychoanalytic current which favored conscious ego functions at the expense of the unconscious ego and id (Rosner 2014a, b). And in fact, even as late as 1970, Beck published an article on the cognitive triad in a psychoanalytic journal (Beck 1970a) and described his model as an integration between neo-Freudian and behaviorist concepts, in which the cognitive triad of CT played a mediational role between trigger and behavioral response (Beck 1970b). In those same years, Beck explored the role of fantasies and dreams in psychology (Beck 1970c, 1971).

## Self-Knowledge in REBT: Not so Core an Irrational Belief

Like Beck, Ellis had strong psychodynamically oriented training, specifically with Karen Horney at the American Institute of Psychoanalysis in New York in the late 1950s (Ellis 1962). Ellis, however, divorced himself from his psychodynamic background in a more rapid and seemingly painless way than Beck. Perhaps this allowed him to be less influenced by self-knowledge related concepts. In Ellis’ REBT model, emotional distress depends on maladaptive evaluations called “irrational beliefs” which share much less Beck’s CT focusing less on the self and self-knowledge. This is an important difference between REBT and all other cognitive therapies (Ellis 1962; Ellis and Grieger 1986).

In its most recent formulations, REBT focuses on four types of irrational beliefs: “demandingness”, “awfulizing”, “frustration intolerance”, and “self/other worth ratings” (DiGiuseppe et al. 2014, pp. 34–36). Among these beliefs, self/other worth ratings is probably closest to a self-knowledge structure. However, the role of self/other rating in REBT is different from the mechanism played by self-beliefs in either Beck’s CT or other CBT models for various reasons. First, self/other worth ratings is not the pivotal mechanism in the psychopathological model of REBT. In REBT the core role is played by demandigness, while the other three mechanisms could not work if not triggered by demandingness and are therefore called derivative irrational beliefs (DiGiuseppe et al. 2014, pp. 36–39). Second, self/other worth ratings are not related only to the self. Other people can be the object of the evaluations as well as the self. Third, while CT’s biases encompass the whole chain of cognitive inference triggered by initial automatic thoughts, REBT’s irrational beliefs are restricted to the final, most upsetting, evaluative step. In Beck’s CT model, the chain of negative inference starts from the initial automatic thought (e.g., “this performance is difficult”), evolves in subsequent assumptions (e.g., “if it is difficult, I may fail”), and ends in negative self-beliefs (e.g., “I am a failure”). REBT focuses on an evaluative step which comes after the chain of inferences. The evaluation makes the final result irrational. For example, in case of awfulizing (“if I fail it’s awful”) the irrational step that creates the emotional disorder is the final awfulizing evaluation: “if I fail *it’s awful*,” and not the preceding chain of negative thoughts, “*if I fail*.” Failure is a possible scenario in our daily life: who can take success for granted?

This difference between CT and REBT is very telling when applied to “self/other worth ratings” where the psychopathological problem is actually not related to the specific content of a self-belief. Many beliefs about the self will be neither wrong nor irrational, given that a person may be actually perform poorly or lack skills or abilities. But the additional global evaluation of his or her worth as a person will always be irrational, for there is no agreed upon standard or science or logic to human rating. In fact, in REBT “self/other worth ratings” is not replaced by empirically based positive self-ratings but by the functional “unconditional self acceptance” (USA) in which self worth is not related to performances and self-judgments but it is recognized as an intrinsic attribute of human dignity (DiGiuseppe et al. 2014, pp. 50–54).

Summing up, in REBT, emotional disorders do not depend on a structurally biased self knowledge but on functionally maladaptive evaluations which are only partially related to self knowledge. This suggests that REBT is not only the first version of “second wave” cognitive therapy but also a forerunner of the emerging, functionalistic “third wave” switch in clinical cognitive paradigm.

## Self-Knowledge in Beck’s CT as a Core Belief

It might also be true that concept of self-knowledge helped Beck to formalize his procedures in amenable ways for clinicians, who perhaps found self-beliefs more understandable and manageable than abstract cognitive biases. Moreover, Beck’s crucial advantage was his allegiance to the development of replicable protocols applicable to the DSM system of psychiatric diagnoses. This strategy allowed Beck to perform the first true randomized trial that proved CT’s effectiveness for the treatment of depression (Rush et al. 1977).

A final breakthrough came with application of the Beck’s CT model to anxiety disorders, grounded in research from the University of Oxford led by Clark and Salkovskis. They worked out a series of cognitive therapy protocols modelled on the work of Beck and applied to a wide range of psychological disorders: panic disorder (Clark 1986), social phobia (Clark and Wells 1995), post-traumatic stress disorder (Ehlers and Clark 2000), eating disorders (Fairburn et al. 1999) and obsessive–compulsive disorder (Salkovskis 1985). Importantly, the clinical efficacy of these protocols was demonstrated in controlled studies (Clark et al. 1999; Hollon and Beck 1994; Hollon et al. 1996, 2006; Nathan and Gorman 2015; Otte 2011).

The British academics borrowed from Beck both his “psychodynamic” care for verbal reattribution focused on self-beliefs and his “psychiatric” attention for DSM diagnoses (Rachman 2015). However, they strongly reintroduced the behavioral element which has historically been central in the British landscape, based on the work of Victor Meyer in the days of the Protocol for Exposure and Response Prevention (ERP) for obsessive compulsive disorder (Meyer 1966). British behaviorism was more likely to provide a suitable candidate for merging with Beck’s CT because of Meyer’s efforts in developing appropriate case formulation procedures (Bruch 2015; Marks 2012; Rachman 2015). Beck also expanded the range of applications and increased behavioral components in his model (Beck et al. 1979, 1985).

Thus, a standard clinical model was born, called CT in the USA and CBT in the UK (not to be confused with the use of the term “CBT approaches” to indicate the total set of all cognitive behavioral therapies). The standard model had the fundamental tenets that emotional disorders depend on biased automatic cognitive processes which can be changed through verbal reattribution in therapy (Beck 1976; Clark et al. 1999; Clark and Beck 2010; Dobson and Dozois 2010; Ellis and Grieger 1986; Goldberg 2001; Kazdin 1978; Kelly 1955; Mahoney 1974; Meichenbaum 1977; Rachman 1977).

## Self-Knowledge in Constructive Cognitive Therapies

Although it is not true that Beck and Ellis are the only theorists to deserve praise for the development of the basic clinical model of CBT approaches, it is true that today they receive much more attention than the likes of Lazarus, Mahoney, and Meichenbaum.

In the past the scenario was different. In addition to Beck’s CT and Ellis’ REBT the range of CBT approaches also included *Covert Sensitization* (Cautela 1967), *Problem*-*solving and Behavior Modification* (D’Zurilla and Goldfried 1971), *Multi*-*Modal Behavior Therapy* (Lazarus 1976), *Cognitive Behavior Modification* (Meichenbaum 1977), *Clinical Behavior Therapy* (Goldfried and Davison 1976), *Systematic Rational Restructuring* (Goldfried et al. 1974), *Post*-*rationalist Cognitive Therapy* (Guidano 1991), *Constructive Therapy* (Mahoney 2003) and others.

Summing up, in the decade 1971–1980 the clinical models of these scholars occupied the scene with authoritativeness equal to Ellis’ REBT and Beck’s CT, as also suggested by the number of Google citations. Table 1 reports the results of a Google Scholar citation search in which we entered the terms “Albert Ellis”, “Aaron Beck”, “Arnold Lazarus”, “Donald Meichenbaum”, “Michael J. Majoney”, “Marvin Goldfried”, “Vittorio Guidano”, “Gerald Davison”, “J.R. Cautela”, and “Thomas D’Zurilla” for each reported decade from 1951 on. The table shows how in the decade 1971–1980 the influence of Lazarus, Mahoney, and Meichenbaum was still comparable to that of Beck (while Albert Ellis seems to play in another league), at least in terms of Google Scholar citations. The scene changes from 1981 onward: Beck’s influence became closer to that of Ellis while the others were left behind (Table 1).

**Table 1:** Google Scholar citations of major clinical theorists of cognitive behavioral therapy

	1951–1960	1961–1970	1971–1980	1981–1990	1991–2000	2001–2010	2011–2018
Albert Ellis	328	610	1060	1300	1890	4500	5290
Aaron Beck	25	33	209	399	915	3020	4650
Arnold Lazarus	23	164	262	272	349	585	617
Donald Meichenbaum	–	14	153	201	296	470	609
Michael J. Majoney	–	7	192	156	190	219	135
Marvin Goldfried	3	32	86	90	142	215	186
Vittorio Guidano	–	–	7	19	71	239	283
Gerald Davison	–	35	77	47	84	115	73
J.R. Cautela	7	54	108	78	69	75	38
Thomas D’Zurilla	–	7	13	16	28	49	30

Among them, the preeminent figures were Lazarus (1976), Mahoney (2003), and Meichenbaum (1977). While Meichenbaum applied his model mainly to social and community psychology, Lazarus and Mahoney were the scholars who devoted their major theoretical efforts to the development of a clinical model comparable to Ellis’ and Beck’s (Dobson and Dozois 2010). Unlike Beck and Ellis, however, Lazarus and Mahoney were not trained psychodynamically. From a certain viewpoint, they were the authentic clinical counterparts of the non-clinical theorists of the cognitive revolution; those who, coming from a behaviorist background, applied the cognitive revolution in the clinical field and promoted CBT approaches. Lazarus and Mahoney were also the scholars who, during the 1970s, diverged from their original behaviorism and imagined that it was possible to design therapeutic procedures focused on mental content and not on the behavioral trigger-response. A similar theoretical effort was dealt with by Goldfried (1971) who described systematic desensitization in terms of a general mediational model, in contrast to Wolpe’s (1958) counterconditioning model. Moreover, Mahoney was the person who actually favored the general acceptance of the term “cognitive” by establishing in 1977 the eponimous journal “Cognitive Therapy and Research” with himself as inaugural editor (Dobson and Dozois 2010).

Basically, Lazarus and Mahoney were unsatisfied with the behavioral techniques and felt it was necessary to introduce into the behavioral model a cognitive mediator on which it would possibly verbally intervene via the conscious channel. It is no secret that the channel of conscious thought was devalued not only by psychoanalysis, but also by behaviorism. Not only psychoanalysis, but also behaviorism conceived mental suffering as a dysfunctional state learned in a state of unconsciousness, either through unconscious drives or non-conscious, behavioral conditioning. The mind was reduced to a zombie driven by unconscious forces (Liotti and Reda 1981). Lazarus and Mahoney seemed to feel a need for a more psychological model of mental suffering, which would recognize the role played by conscious thought (Lazarus 1977; Mahoney 1984, 1991). But before Ellis and Beck there was a singular blindness in the theoretical perspective of behavior therapists to the patient’s psychological life, whose emotions and experiences might never be directly addressed in the session. At least, this was the theoretical perspective. On the other hand, an anecdotal communication by an expert behavior therapist who practiced in those days tells a different story: thoughts were investigated calling them rule governed behaviors (Mosticoni 2018).

However it is also true that Mahoney retained from behaviorism the awareness that cognitive processes cannot be reduced to their conscious representations in terms of internal dialogue, as Beck and Ellis did. Mahoney considered this development an oversimplification and posed the need for a more sophisticated definition of cognition at the ground of the notorious and self-defeating distinction between a “rationalist” and a “constructivist” approach (Mahoney 1995b, p. 7). The rationalist approach viewed cognition as a direct appraisal of reality immediately accessible to consciousness. The constructivist approach conceived of cognition as a hermeneutical and proactive activity developing not only in conscious terms but also in terms of “tacit” knowledge (Mahoney 1995b; Guidano and Liotti 1983; Guidano 1987, 1991).

Mahoney’s theoretical development toward constructivism was encouraged by his encounter with other constructivist theorists during a sabbatical mainly spent in Europe at the end of the 1970s. In particular he started cooperating with Vittorio Guidano and encouraged his publications (Guidano and Liotti 1983; Guidano 1987, 1991). In addition to the front-runners Guidano and Mahoney, many other authors contributed to development of the constructivist branch of cognitive therapies (Balbi 2004; Feixas and Miró 1993; Guidano and Liotti 1983; Guidano 1987, 1991; Guidano and Quiñones 2001; Lorenzini and Sassaroli 1995; Mahoney 1974, 1991, 1995a, b, 2003; Muran and Safran 1993; Neimeyer 2009; Neimeyer and Mahoney 1995; Winter and Viney 2005). From a clinical viewpoint, constructive therapists preferred interventions focused on personal meanings, including reconstruction of the patients’ life stories and treatment of recursive vicious circles of discomfort with emotion and fear of fear. This intervention anticipated metacognitive concepts, in a way akin to REBT’s concept of secondary ABC (Sassaroli et al. 2005).

In the long run, constructivism indulged in highly speculative thinking which risked not addressing empirical challenges. This propensity for speculation fell under the influence of Maturana and Varela (1980) and von Glaserfeld (1995), went hermeneutic and radically constructivist, and rejected the development of the replicable “constructivist” treatment protocols and case formulation procedures based on DSM diagnoses that were standard features of Beck’s CT and arguably its major strength (Guidano 1991; Mahoney 2003; Neimeyer 2009).

However, at the beginning of the 1980s, theoretical sophistication gave a temporary prevalence to the constructivist branch, given that even Beck and Ellis proclaimed themselves constructive therapists for a while (Mahoney 1995b, pp. 6–10). The constructivist branch considered the Beckian style of verbal assessment and reattribution of beliefs to be simplistic and CT was viewed as affected by a form of crude computationalism inapplicable to the complex fluidity of mental reality. Constructive therapists preferred to talk about personal meanings, referring to Bruner (1973) and, to a lesser extent, to Kelly (1955). Unlike beliefs, personal meanings would be more closely tied to the personal life history of the patient and to his or her emotional experiences. Personal meanings were not a single set of beliefs about a situation, but a vision of the self and the world (Mahoney, 1995b, pp. 11–13).

## From Self-Knowledge to the Therapeutic Relationship

Despite all the possible theoretical differences between “rationalist” and the “constructivist” therapies, it must noted that constructivist therapies also conceived of *self*-*knowledge* and *self*-*schemata* as having a super- ordinate structure that explained both healthy and psychopathological states. This is true not only for Mahoney and Guidano, but also of Kelly’s personal construct therapy, as developed by his epigones (Neimeyer and Mahoney 1995; Winter and Viney 2005; Neimeyer 2009). Moreover, “rationalist” and “constructivist” approaches converged toward a structuralist conception of self-knowledge and self-schemata that developed into a sort of nosography of personality in both opposing camps. In 1993, DiGiuseppe and Linscott have provided evidence indicating that rationalism and constructivism are not bipolar philosophies and there is no evidence that these philosophical difference reflect actual differences in they way therapists conduct therapy. It is remarkable that even within the Beckian CT tradition—especially with Judith Beck’s compendium works (1995, 2005)—we can observe the emergence of a classification table of self-beliefs that ends resembling the constructivist architecture of personality organizations outlined by Guidano and Liotti (1983) and confirmed by Mahoney (Mahoney et al. 1995; Mahoney 2003). It is plausible that the emphasis—increasing over time—on self-beliefs and self-schemata within CT may have been influenced by the attention paid to the concept of personality organization in the constructivist branch of the cognitive therapies (see Table 2).

**Table 2:** Self-beliefs in cognitive behavioral therapy and in constructive models

Cognitive Behavioral Therapy Self beliefs (adapted from Beck 2011, p. 233)	Constructive personality organizations (Guidano and Liotti 1983, pp. 171–306; Mahoney et al. 1995; Mahoney 2003)
*Helpless self* Defective; Failed; Helpless; Incompetent; Ineffective; Loser; Needy; Not good enough; Out of control; Powerless; Trapped; Victim; Vulnerable; Weak	*Phobic personality organization* Being despised; Being ridiculed; Needing protection; Not amiable; Not in control; Unable to cope with; Weak
*Unlovable self* Bad; Bound to be abandoned; Bound to be alone; Bound to be rejected; Defective; Different; Unattractive; Uncared for; Undesirable; Unlikeable; Unlovable; Unwanted	*Depressed personality organization* Abandoned; Being wrong; Disappointed; Failed; Helpless; Isolated; Missing significant ones (loss); Needing approval; Not loved; Rejected; Separated; Worthless
*Worthless self* A waste; Dangerous; Don’t deserve to live; Evil; Immoral; Toxic; Unacceptable; Worthless	*Obsessive personality organization* Controlled; Detached; Doubtful; Guilty; Judgmental; Looking for certainty; Moral; Perfectionistic; Responsible; Restrained; Unemotional
	*Eating disordered personality organization* Adhering to other ones’ judgment; Craving for emotional contact; Dependent; Self-criticizing; Self-deprecating; Undefined

In turn, growing attention to self-knowledge and self-schemata may have depended upon an increasing ‘contamination’ of theoretical backgrounds rooted in both the psychodynamic and humanistic fields. Many clinical and non-clinical theorists have developed models in which the self plays a structural role in organizing, coordinating, and regulating the other psychological functions. The self provides consistency, continuity, and identity in the individual development; it is not the result of memory and information but is the guarantee of good psychological functioning. Albeit with some differences depending on their approach, we could mention not only Erikson (1950), Kohut (1977), and Rogers (1977), but also Bandura (1977) as examples of a trans-theoretical emergence of the self as organizing structure around which individuals may build their psychological wellbeing. Of course, Bandura’s self-efficacy and Erikson’s self-identity show many different features. However, it cannot be denied that, from the 1970s onwards, the self was a landing place where scholars with backgrounds as different as Bandura, Erikson, Kohut, and Rogers, found a meeting spot in which their theoretical distances significantly decreased. The self was a good alternative to the lost explicative power of concepts like Freudian drive or Beckian beliefs.

In turn, it should be stressed that the parallel movement went on even after the discovery of the self in these theories. In fact, the prevalence of the self ended up generating an increasing attention towards the therapeutic relationship in many therapeutic approaches. From the 1980s on, the theoretical development of most therapeutic approaches saw the appearance of models focused on the analysis of interpersonal patterns in the direct experience and management of the therapeutic session. This evolution is observable in both standard CBT (JS Beck 2005, pp. 15–16; Hofmann et al. 2013; Leahy 2008, 2015) and in the constructivist therapy (Dimaggio et al. 2007; Hermans and Dimaggio 2004; Mahoney 2003; Neimeyer 2009).

A similar movement was even more pronounced in the humanistic and the psychoanalytic fields, which were, historically, already interested in relational aspects. Rogers had paid attention to the relationship from the beginning of his development of Client Centered therapy (Rogers 1959) while in psychodynamic theory the interest showed a further step ahead when the so-called relational paradigm (Mitchell 2000) emerged. Summing up, we may consider this centrality of the therapeutic relationship to be the natural development of a theoretical conception that offers a key-role to self-structures.

## Back to Functionalism?

In contrast with the convergence toward structuralism and self-schemata of both CT and constructivist therapies, it would also be possible to interpret the so-called “third wave” clinical models as a back-to-functionalism theoretical movement and an anti-structuralist reaction (Hayes and Strosahl 2004; Martell et al. 2001; Kanter et al. 2009; McCullough 2003; Linehan 1993; Kohlenberg et al. 1993; Christensen et al. 1995). “Third wave” CBT approaches are characterized by a heterogeneous array of themes: acceptance, experiential intervention, cognitive fusion, commitment, compassion, developmental aspects, dialectics, emotions, metacognition, meditation, mindfulness, while more broadly it also involves interpersonal themes, therapeutic relationship, and spirituality (Kahl et al. 2012). In these models, the importance attributed to self-knowledge and self-schemata is sharply reduced, while the therapeutic process focuses on either *top down* mental functions such as voluntary attention and executive control governed by metacognitive procedures or on *bottom*–*up* experiential and interpersonal processes. The role played by these mental functions may be an indicative criterion for understanding the new directions taken by these models with respect to early CT and constructivist models.

In Beck’s CT and in constructivist models voluntary attention and executive control depend on the elaboration of cognitive contents related to self-knowledge and self concepts. These models hypothesize that the therapeutic mechanism depends on so-called “first-order change” that is change by direct exploration and modification of cognitive evaluations of external reality without any second level action on internal meta-cognition, e.g. beliefs over emotions, behaviors and even over other beliefs (Lyddon 1990; Wells and Mathews 1994, p. 2). However, this is exactly the place where theoretical doubts about “second wave” models have focused their criticisms. Empirical research, despite having found correlations between cognitive changes and decreased emotional distress (Burns and Spangler 2001; Morgenstern and Longabaugh 2000), has failed to conclusively prove that the effectiveness of either Beck’s CT or other CBT approaches -including constructivist models- depended on a “first-order change” (Dobson and Khatri 2000, p. 913; Hayes 2004; Illardi and Craighead 1994; Jacobson et al. 1996). There was a lack of evidence about the supposed direct relationship between the mental representations of self-knowledge and the architecture of emotional and behavioral dysfunctional processes (Mathews and Wells 1999, p. 180; Rosenfarb and Hayes 1984). It is no coincidence that in 1996 Beck, himself -in paradoxical concurrence with the mundane success of his CT model- published a paper entitled “Beyond belief” (Beck 1996).

The new, “third-wave” models propose that emotional disorders do not depend on mental representations of the self (again, self-knowledge and self-beliefs) as Beck thought (Beck 1976), but on dysfunctional regulation of the interaction between voluntary processes—first of all attention and executive control- and automatic, and emotionally-laden associative processes (Kahneman and Frederick 2002; Martin and Sloman 2013; Sloman 1996, 2002; Stanovich 1999; Stanovich and West 2002; Wells and Mathews 1994).

**Fig. 2:**
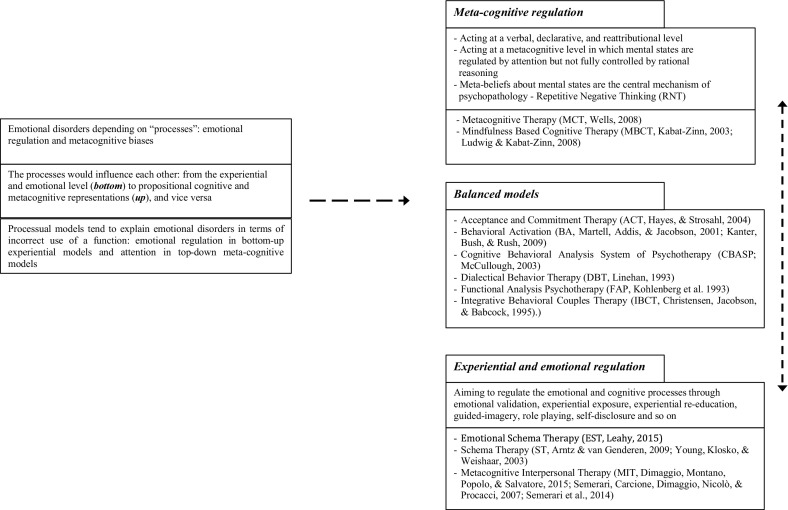
Process oriented therapies: a possible classification

Actually, these processes are not rigidly separated but influence each other in many directions which can roughly be reduced to a *bottom*–*up* direction, from the sensory-motor level perceived as emotions (*bottom*) to voluntary propositional cognitive and metacognitive representations (*up*), and vice versa, that is *top*–*down*. In turn, this bi-directional reductionist model may be also useful in order to map therapeutic interventions: *bottom*–*up* interventions that aim to regulate the emotional and cognitive processes through experiential exposure, experiential re-education, guided-imagery, or role playing (Bell et al. 2015; Hackmann et al. 2011); and *top*–*down* interventions aiming at acting at a verbal, declarative, and re-attributional level. *Top*–*down* interventions are, however, implemented mostly at a second-order metacognitive level in which mental states are regulated by attention, but not fully controlled by rational reasoning (Wells and Mathews 1994; Williams et al. 1988). Figure 2 visually represents this map.

The detail that *top*–*down* interventions operate at a metacognitive level in which mental states regulates other mental states and not at a direct cognitive level in which beliefs evaluates external reality helps us remember that processing interventions are complex and not reproduce the simplistic opposition between rationalism and constructivism: in each of them, next to the *bottom*–*up* experiential component, there is always a *top*–*down* verbal reattribution component, and vice versa. Moreover, some models show an additional interpersonal component focused on the therapeutic relationship and on the assessment of the personal life history of the patient. We may classify the new models in different groups in terms of proclivity towards one or the other poles of attentional controllability: from high levels of metacognitive controllability to less controllable levels of experiential change. Some models appear as balanced while others are very prone to privilege one of the poles.

## Balanced Models: “Third Wave” Therapies

A first group are the therapies strictly belonging to the “third wave”: *Acceptance and Commitment Therapy* (ACT, Hayes and Strosahl 2004), *Behavioral Activation* (BA, Martell et al. 2001; Kanter et al. 2009), *Cognitive Behavioral Analysis System of Psychotherapy* (CBASP; McCullough 2003), *Dialectical Behavior Therapy* (DBT, Linehan 1993), *Functional Analysis Psychotherapy* (FAP, Kohlenberg et al. 1993), and *Integrative Behavioral Couples Therapy* (IBCT, Christensen et al. 1995). Basically, these models have maintained a strong relationship with the behavioral tradition and represent a return to contextualism and functional analysis (Jacobson et al. 2001). However, while traditional behavioral therapies focused heavily on change, “third wave” approaches attempt to integrate the twin goals of acceptance and change as positive outcomes. They encourage both the development of mental flexibility in managing either adverse situations or emotional discomfort and the promotion of a larger behavioral repertoire in daily life. Among these approaches, ACT is the most popular. Its main focus is to achieve a mental state of both accepting flexibility and commitment to personal values. In addition, ACT frequently shows a sophisticated experiential component that resembles an updated form of behaviorist extinction, but includes a more expanded degree of metacognitive awareness (Hayes et al. 2013). Similar considerations can also be made for CBASP and FAP, which also pursue behavioral flexibility. In general, all “third wave” approaches recommend flexibility in balancing acceptance of symptoms and change; these are, in turn, grounded on the Skinnerian distinction between contingency-shaped and rule-governed behavior (Skinner 1966, 1969).

Summing up, flexibility, acceptance and commitment to change in third wave therapies should be not confused—despite the similarity—with any self-knowledge related concept. In fact, these models conceive flexibility as action related and rule governed behaviors and not as an internal knowledge about the self (Cordova and Eldridge 2000; Hayes and Strosahl 2004; Hayes et al. 2013). In terms of either top down or bottom up processes, “third wave” models have a balanced approach given that they both encourage a higher top down flexible increase of the function of conscious executive control, although essentially in terms of acceptance and commitment to change; however, “third wave” models also consider essential the experiential bottom up path, given that any conscious increased degree of flexibility is conceived as grounded in a experiential reinforcing context of reinforcing behavioral improvements.

## Top–Down Models: Metacognition and Mindfulness

While core “third wave” therapies are balanced, prevalently *top*–*down* interventions assign an active role to the explicit mental representation and willing control—albeit metacognitive—of processes. The metacognitive biases are not distorted patterns of reality evaluation in Beckian CT style but beliefs on the mental activity itself, i.e. dysfunctional meta-representations. Admittedly, the importance of metacognitive components had already been intuited by Beck himself when he described the vicious circles of fear of fear (Beck et al. 1985) or even more sharply by Ellis with his seminal concept of secondary ABC (DiGiuseppe et al. 2014, pp. 64–65). Moreover, even constructivist approaches conceptualized the secondary ABC as an intervention that foreran metacognitive concepts (Sassaroli et al. 2005). All in all, it is not coincidental that Windy Dryden called the secondary ABC a ‘meta- emotional problem’ (2011, p. 70), a name emphasizing its affinities with metacognitive models. In fact, the secondary problem is a biased irrational belief that patients have towards their mental states. Such a development is also observed in the CBT field with Leahy’s Emotional Schema Therapy (EST, Leahy 2015), a therapeutic model that, while not denying Beck’s CT filiation, focuses on meta-emotional schemata, namely beliefs about emotions as major pathological mechanisms and therapeutic targets: A brilliant way to move from a structuralist self-schema model to a more updated process oriented functionalism.

However, the new development is that, in the metacognitive models, it is firmly held that metacognitive beliefs about mental states are not only possible psychopathological biases among others, but are the central mechanism of psychopathology and the core theoretical principle (Mathews and Wells 1999, 2004; Wells and Mathews 1994; Wells 2008, 2013). These dysfunctional metacognitive beliefs are activated when the person in distress reacts to emotional discomfort—transforming it in an emotional disorder—by activating repetitive cycles of so-called *Repetitive Negative Thinking* (RNT) that feed on themselves for two main reasons: they are mistakenly conceived as functional plans for coping with reality and its problems (Gross 2002; Hayes and Feldman 2004; Salovey et al. 2000; Segerstrom et al. 2003; Wells 2008) and/or are deemed as an uncontrollable state stronger than personal executive willingness (Mathews and Wells 1999, 2004; Wells 2008, 2013; Wells and Mathews 1994; Williams et al. 1988).

There are several different treatment protocols which give importance to metacognitive factors: *MetaCognitive Therapy* (MCT, Wells 2008, 2013), some components of the above mentioned ACT (Hayes and Strosahl 2004) and many mindfulness-based interventions, among which the most developed is *Mindfulness Based Cognitive Therapy* (MBCT, Kabat-Zinn 2003; Ludwig and Kabat-Zinn 2008). All these models emphasize the possibility of managing dysfunctional processes through training aimed to distance oneself from painful and worrying mental states, albeit it is well known that any mindfulness practice also recommend to pay attention to the *bottom up* aspect of mental states.

Among all these approaches MCT more than any other considers metacognitive function as the center of psychopathology and ignores any hint to self schemata, self concepts, and self structures. In MCT metacognition is neither a structure nor a frame that holds everything together. Were metacognition a structure, we would return to a self-psychology, albeit reformulated in metacognitive terms: a meta-self psychology! Metacognition is rather a function that performs certain tasks in certain moments of mental life and that is always at risk of being used in dysfunctional and anti-economic ways, hence generating emotional disorders (Wells 2008, 2013). MCT works using a specific case formulation and therapeutic project treating metacognitive dysfunctions that had previously dysregulated patients’ attention and hindered the development of his or her environmental adaption. Summing up, attention and metacognition are the functions on which MCT specifically acts (Wells and Mathews 1994, pp. 20–23).

## Bottom–Up Models: Emotions, Experiences and Residual Self-Knowledge

Among prevalently *bottom*–*up* models we can list two therapeutic protocols which integrate experiential interventions and process oriented, developmental and interpersonal components and preserve a clinical and theoretical interest in self-knowledge structures. Actually, their residual interest in self-knowledge is not coincidental given that the two models are later developments of the early standard cognitive and constructivist models respectively. They are *Schema Therapy* (ST, Arntz and van Genderen 2009; Young et al. 2003), a model primarily developed from a CBT clinical and theoretical background, and *Metacognitive and Intepersonal Therapy* (MIT, Dimaggio et al. 2015; Dimaggio et al. 2007; Semerari et al. 2007; Semerari et al. 2014) which can be considered a development of the constructivist model of Guidano and Liotti (1983) and Mahoney (2003). Also above mentioned Leahy’s EST could be included in this group.

ST preserves a strong interest in self-knowledge. In fact -as its name says- ST conceptualize emotional disorders in terms of biased self-schemata which however are not only purely cognitive as in Beck’s CT but also shows a strong emotional and interpersonal aspect rooted in the personal development of the patient (similar developments as also present in Leahy’s EST). These interpersonal features are represented in so-called “modes” which are stereotyped and inflexible interpersonal patterns. However, there is also a metacognitive and functional component in the “modes” because modes’ stiffness depends on a state of cognitive fusion between patients and their active “modes” (Arntz and van Genderen 2009). There is therefore a *top*–*down* aspect, however, immediately denied by ST’s therapeutic style, which is largely *bottom*–*up*, because in ST the therapeutic change is conceived as happening through an intense corrective emotional experience in which the painful events that underlie dysfunction are relived in a non-traumatic way (Young et al. 2003).

In the MIT model the emotional pain would depend on the metacognitive deficits in the skills to identify emotions, to interpret our own mental states, to distinguish them from those of others, and finally to behaviorally master them (Semerari et al. 2007; Semerari et al. 2014). It is a complex multi-function that somehow includes either *top*–*down* or *bottom*–*up* processes. However, the therapy appears to prefer mainly *bottom*–*up* techniques of emotional and relational adjustment by encouraging the observation and appreciation of the most minute details of daily life and reality, in order to overcome the tendency to worry, ruminate and produce over-interpretations (Dimaggio et al. 2015). After this experiential phase, MIT stimulates the development of higher metacognitive functions. The therapeutic relationship is conceived as an in vivo opportunity to experience this type of complex thinking (DiMaggio et al. 2007). However, it is prescribed that the final step of the therapy would include the construction of more positive self-beliefs. Therefore, MIT retains some structural self-knowledge related concepts (Dimaggio et al. 2015).

In these protocols the *bottom*–*up* route is privileged. The prevailing idea is that the experiential intervention, mostly bodily and, in some cases, relational—always precedes any conscious emotional regulation, which comes only at a later time to lay down skills learned via the body in a new cognitive routine. Therapy is basically an emotional and relational experience where new regulative skills are never learned consciously by executive control (Liotti 2001).

## Conclusion: Not an Affiliation Story, but Uneven Turning Points

The bottleneck-regulating function of either metacognitive processes or experiential interventions is a strong alternative to the structuralist self-psychology of the “second wave” CBT approaches. It is not a direct development, according to a filiation storytelling that would naturally go from behaviorism to the cognitive revolution. In this paper we have tried to disconfirm the hypothesis in which every historical turning point is fully consistent with its historical roots planted in earlier times. We have shown that clinical models are not the mechanical application of the cognitive revolution to therapy, having cognitive therapies also received the waters of a psychodynamic tributary (Rosner 2014a, b), and we have also shown that this tributary has helped to give a structuralist character of self-psychology to cognitive therapies. This structuralist character was already in sharp break with the functionalist nature of behaviorism.

In turn, also the process oriented turning point denies the structuralism of the self-schema theory which affected CBT approaches, either Beck’s CT or constructivist. Process oriented models do not explain emotional disorders in terms of a hidden broken structure, the self, but to the incorrect use of a function: attention in *top*–*down* metacognitive models, emotional regulation in *bottom*–*up* experiential models. This functionalist conception brings process oriented models closer to some behaviorist concepts than to clinical cognitive schema theory, because they proposes a renaissance of “first wave” principles such as extinction reinterpreted from a metacognitive viewpoint. Perhaps, a sort of mentalist and metacognitive variation of both Pavlov’s classical respondent conditioning (1927/1960) and Skinner’s operant conditioning (1954).

The hypothesis of the behavioral root of the metacognitive model has a further confirmation. The metacognitive model, before being brought to maturity by Wells, had found its main precursor in Tom Borkovec’s studies about insomnia and then generalized anxiety disorder (Borkovec and Inz 1990; Borkovec et al. 1998). Borkovec explored insomnia and anxiety not in terms of cognitive beliefs, but in relation to functional processes, such as worry and brooding, in turn dependent on metacognitive beliefs: “worry helps me coping and therefore I do it, even if it costs me sleep and peace of mind”. What matters was that Borkovec had a never repudiated behaviorist background. So he considered anxiety more like a behavior equipped with its function that as an underlying cognitive structure. For this reason Borkovec ended up investigating worry as a behavior of the mind and a process, but not a belief (Borkovec 1994).

Of course, this does not mean that in classic cognitive models process oriented aspects where not represented or prefigured. As written above, when Beck described the role played by vicious circles of fear of fear (Beck et al. 1985) and when Albert Ellis conceived the influential concept of secondary ABC (DiGiuseppe et al. 2014, pp. 64–65) heralded functionalist metacognitive processes. Moreover, REBT’s functionally maladaptive evaluations foreshadowed the functionalistic switch of the “third wave”.

In addition, there are also elements that make metacognitive and behaviorist models different from one another. Among them, the main difference appears to be the non contextualist setting of Wells’ metacognitive model. In fact, the metacognitive model selects a *bottleneck* on which to surgically act to obtain the maximum therapeutic effect. Basically, this strategy is anti- contextualist, and makes the metacognitive model very different from the more recent contextualist developments of behaviorism, like the ACT (Hayes 2004).

In conclusion, what really matters in the process oriented models and above all in metacognitive models is the innovative conception of cognitive function. No longer a holistic mediation, a sort of allmighty homunculus intermediate between trigger and response, but, as already highlighted, a metacognitive ‘retroactive’ executive control (Wells and Mathews 2015, p. 31) even when it is learnt in experiential *bottom*–*up* interventions. This theoretical turning point allows to reformulate the clinical cognitive theory in more promising terms and encourages clinicians and patients to increase the level of awareness and knowledge of how voluntary functions of cognitive control and attentional selection of information really work. This can be translated into new effective strategies of retroactive detached and mindful management of suffering states in emotional disorders.

In addition, process oriented models also would allow to conceive in a new and different way the relationship between cognitive therapies and behaviorism, forcing all of us to rethink both behaviorism and cognitivism in less simplistic terms. Behaviorism, although perhaps maintaining an insurmountable idiosyncrasy for mentalistic functions, cultivated within it the fruitful idea of functionalism and feedback control. Cognitivism still retains its revolutionary value not so much in the idea of cognitive mediation, but in the importance it gave to the function of executive control and voluntary mental representation.

However, it is perhaps true that the new models, and perhaps especially the metacognitive ones, sometimes can be tricky for the clinician who may not be very interested in the theoretical sophistication of debates about functionalism versus structuralism. Process therapies, although scientifically robust, may damage the communications between theorists and clinicians and may decrease the intuitive clinical simplicity of “second wave” CBT approaches, including Beck’s CT. In addition, the clinician has a natural tendency to formulate the case in terms of a narrative of the self rather than in impersonal functions. In order to satisfy this need it will be beneficial to propose functionalist models compatible with the narrative mindset of clinicians; models that would contain both the developmental ground of the disorder, the narration of the painful experiences that transformed them in emotionally vulnerable individuals, and the mental processes that patients have mistakenly cultivated deeming them functional or uncontrollable, on the ground of dysfunctional metacognitive beliefs (Wells 2008).

Last but not least, despite all the innovations offered by process therapies, it must be stressed that from an evidence-based viewpoint Beck’s CT maintains the strongest and most solid effectiveness results for many emotional disorders in the area of depression, anxiety and eating disorders (Nathan and Gorman 2015). None of the new therapies have shown better results in the target area of CT, namely depression, anxiety and eating disorders, with the exception of MCT which in a recent meta-analysis resulted significantly more effective than both waitlist control groups as well as CBT (Normann et al. 2014). On the other hand, it is true that the available evidence now allows considering all third wave treatments as empirically supported as well as CT in some areas—for example, EMDR is as effective as CT for Post Traumatic Stress Disorder—and even superior in other cases, namely DBT for personality disorders (Kahl et al. 2012). Moreover, the reflection about the emergence of process oriented and functionalist models may provide suggestions about the old question that all these therapies have some empirical support. As known, the common answer up until now has been the common factors, a theory that proposes that different approaches in psychotherapy share common factors that account for much of the effectiveness. Among these common factors, therapeutic relationship factors are frequently emphasized (Wampold and Imel 2015). Of course, such an explanation would question the empirical data -based on randomized placebo-controlled trials- in favor of the greater efficacy of CBT approaches for some disorders, mainly anxiety-related disorders. On the other hand, an alternative explanation which would identify the common factor that accounts for their effectiveness in the process oriented metacognitive focus would also preserve room for the specificity of CBT approaches for some disorders (Smits and Hofman 2008), specificity moreover recently confirmed in an updated meta-analysis (Carpenter et al. 2018).

In conclusion, although the new wave of process oriented and experiential therapies is promising, scientific literature confirms that, at the present time, CT is still the most effective psychotherapy for most emotional disorders (Nathan and Gorman 2015). However, it is possible to imagine possible future directions of development and their empirical and theoretical strengths and weaknesses using the *top down*/*bottom up* classification.

The *top down* orientation has its empirical strength in some promising meta-analyses that suggest an increase in psychotherapeutic effectiveness for some emotional disorders when compared to Beck’s CT, the “golden standard” (Normann et al. 2014). They would therefore be a step forward. The theoretical strength is that attention, the target variable of the metacognitive model, is rigorously operationalizable both in its mechanisms of action and in its effects, ensuring empirical consistency both to the theoretical elaboration and to the verification procedures (Mathews and Wells 2004). It is scientific reductionism at its best. On the other hand, this reductionism can be criticized for theoretical and clinical narrowness. To summarize, this direction seems the most responsive for the purpose of strategically identifying the variables most sensitive to therapeutic action. However, it risks being excluded from broader and less immediately fruitful reflections.

At the other end of the axis we find the models that prefer a *bottom up* orientation. The empirical strength of these models is their promising data for the traumatic clinical area (Lancaster et al. 2016). From a theoretical point of view, these models are based on the fascinating models of embodied cognition (Shapiro 2010). These ambitious models attempt to bridge the mind–body gap and show all the pros and cons of their ambition: on the one hand the audacity of a non-reductionist idea of the functioning of the human system, on the other the risk of investigating models that are not easily operationalizable.

From the clinical point of view, *bottom up* interventions could, in turn, be classified into two large groups. The first group includes therapies that use experiential and imaginative interventions that presuppose an explicit agreement with the patient, a sharing of the rationale and an explicit therapeutic alliance. The second group uses interpersonal interventions that precede the explicit alliance and that are focused on states of crisis in the therapeutic relationship that would be unique therapeutic opportunities.

Actually, the two directions are strictly interwoven with each other. The first group of *bottom up* interventions implies a *top down* component, which is the sharing of the rational intervention and its deliberate and voluntary execution; moreover, it could also be added, a *top down* intervention like a Beckian questioning is also an experience and therefore presents a *bottom*–*up* aspect. The second group, on the other hand, presents itself as a non-schedulable corrective emotional experience in the here and now of the therapeutic relationship and seems not directly compatible with a significant *top down* action, at least at the beginning of its implementation. This is perhaps the case of the model of Safran and Muran (2000) focused on detecting alliance ruptures and rupture repairs in session, in which the only *top down* moment is locked up in the retrospective reconstruction of these critical events. It should be emphasized that cognitive-behavioral therapies have never excluded imaginative and experiential intervention (suffice to mention the interest of Albert Ellis in guided imagery or the use of behavioral exposition in the model of Aaron Beck) but they have always inserted these interventions in the frame of the shared formulation of the case and of the rationale of the intervention. In short, it would seem that the development of cognitive behavioral therapies goes more towards the aware (and therefore *top down*) clinical management of the processes in therapy and this *top down* management can include in its rationale *bottom up* experiential and imaginative processes.
